# They look like gold: CITF1 and FIT interaction mediate cross-talk between iron and copper

**DOI:** 10.1093/plcell/koag229

**Published:** 2026-07-30

**Authors:** Sébastien Thomine

**Affiliations:** Université Paris-Saclay, CEA, CNRS, Institute for Integrative Biology of the Cell (I2BC), Gif-sur-Yvette 91198, France

Because they can give and accept electrons under biological conditions, copper (Cu) and iron (Fe) are 2 precious metal cofactors in major biochemical processes such as respiration and photosynthesis. On the other hand, both metals are highly toxic when present as free ions in cells because they can react with oxygen via the Fenton and Haber-Weiss reactions to form reactive oxygen species and cause mis-metalation of proteins requiring other metal cofactors. Even though they have similar redox properties, Cu and Fe can only replace each other in a few reactions. In plants, the best documented example is the substitution of Cu and Zn-dependent superoxide dismutase (CuZn SOD) by Fe SOD under Cu deficiency (Yamasaki et al 2007). In cyanobacteria and algae, cytochrome c6, an Fe-containing protein can replace plastocyanin that uses Cu as a cofactor for the transport of electrons between cytochrome b6f and photosystem I when Cu availability is too low (Merchant et al 2020).

Yet, there is evidence for intensive cross-talk between Cu and Fe homeostasis. Fe deficiency upregulates Cu uptake in Arabidopsis and rice. This is because the Cu uptake transporter genes, *AtCOPT2* and *OsCOPT1* and *7*, are induced by the major Fe-deficiency-responsive transcription factor FIT in Arabidopsis and rice, respectively (Colangelo and Guerinot 2004; Liang et al 2020; Zhu et al 2024). Conversely, Cu deficiency increases Fe uptake (Bernal et al 2012; Sheng et al 2021; Chia et al 2023). Copper deficiency likely causes secondary Fe deficiency by decreasing the activity of Cu-dependent enzymes involved in Fe oxidation (Bernal et al 2012; Xu et al 2022). Transcriptional responses upon changes in Cu and Fe availability are also interdependent but the molecular basis for this cross-talk was so far not fully elucidated.

**Figure 1:**
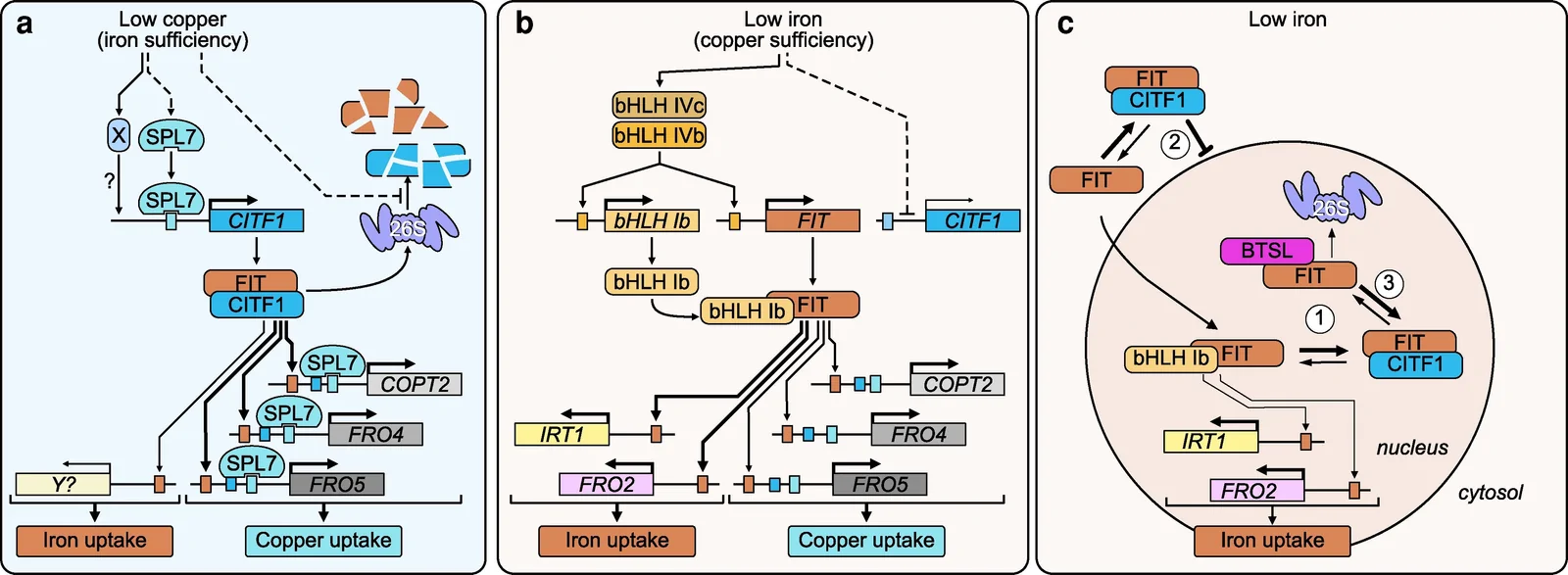
Model for the CITF1-FIT-mediated crosstalk between Cu and Fe homeostasis. a) Under Cu deficiency, *CITF1* is upregulated by SPL7. At the post-translational level, CITF1 physically interacts with FIT to facilitate Cu uptake by upregulating the expression of *COPT2*, *FRO4*, and *FRO5*. Copper deficiency slows proteasome-mediated degradation of CITF1 and FIT, leading to accumulation of the CITF1-FIT complex. b) Under Fe deficiency and Cu sufficiency, CITF1 is transcriptionally downregulated, releasing FIT for subsequent interaction with clade Ib bHLH members. FIT-bHLH Ib interaction promotes *IRT1/FRO2* and *COPT2* expression for proper iron deficiency response. Modified from Chia et al (2026). c) Under Fe deficiency, CITF1 down-regulates *IRT1* and *FRO2* leading to lower Fe uptake, by 2 mechanisms: CITF1 competes with bHLH Ib subunits for binding to FIT, limiting the formation of the active transcriptional complexes FIT-bHLH Ib (1) and association of FIT with CITF1 in the cytosol prevents Fit entry into the nucleus. On the other hand, association of FIT with CITF1 slows down FIT degradation by preventing FIT association with the E3 ubiquitin ligase BTSL (3).

Two recent publications in *The Plant Cell* identified one of the molecular mechanisms underlying the cross-talk between Fe and Cu uptake in *Arabidopsis thaliana* (Cai et al 2026; Chia et al 2026). Both studies show that the transcription factors CIFT1 and FIT, mediating Cu deficiency and Fe deficiency transcriptional responses, respectively, physically interact. This interaction has distinct outcomes depending on Fe and Cu availability (Fig. 1). Chia et al show that, under low Cu deficiency, CITF1 stabilizes FIT and their complex positively regulates Cu deficiency responsive genes. Consequently, the induction of the Cu uptake machinery, including the 2 membrane-bound root Cu^2+^ reductases encoding genes, *FRO4* and *FRO5*, and the transporter encoding gene *COPT2*, is downregulated in a *fit* loss-of-function mutant (Chia et al 2026). In contrast, under low Fe availability, CITF1 negatively regulates root Fe deficiency responses *via* its interaction with FIT. To activate the Fe uptake machinery at the transcriptional level, FIT associates with class Ib bHLH transcription factors, bHLH 38, 39, 100, and 101 to form active complexes (Yuan et al 2008). Both studies show that CITF1 competes with class Ib bHLH for association with FIT. In addition, Cai et al show that association with CITF1 prevents the entry of FIT into the nucleus. Together, these mechanisms lead to decreased expression of the FIT target genes that mediate Fe acquisition, the root ferric chelate reductase gene *FRO2* and the iron uptake transporter gene *IRT1*. Accordingly, *IRT1* and *FRO2* are upregulated in *citf1* loss-of-function mutant and downregulated in CITF1 overexpressing lines. This correlates with higher and lower tolerance to Fe deficiency in *citf1* and CITF1-OE, respectively. Together, the results reported by Chia et al and Cai et al highlight a role of the CITF1-FIT interaction in controlling the balance between Fe and Cu uptake depending on their availability. Although the studies use extreme Cu or Fe deficiency, this regulation likely allows adjustment of the Cu to Fe ratio in a wide range of environmental conditions. Moreover, as FIT is regulated by many other interactions, CITF1-FIT interaction may fine-tune the Cu to Fe ratio to adapt to other environmental constraints leading to oxidative stress.

The studies identify CITF1-FIT as a new node in the intricate network connecting Cu and Fe homeostasis. Previous studies have identified other nodes where this interaction takes place. Iron Man (IMA) peptides that are major players in the regulation of Fe deficiency response are downregulated under Cu deficiency and bind CITF1, decreasing its activity (Cai et al 2024). In addition, the oligopeptide transporter OPT3, which has been implicated in long distance regulation of Fe uptake in Arabidopsis, also transports Cu into the phloem and fine-tunes shoot-to-root Fe/Cu signaling (Chia et al 2023). Finally, the high affinity Cu transporter COPT5 and NRAMP4, a transporter involved in Fe efflux from the vacuole reciprocally affect each other (Carrió-Seguí et al 2019). In future studies, it will be interesting to understand how the different nodes of interaction between Cu and Fe homeostasis integrate. To define more precisely the importance of FIT-CITF1 interaction, it would be interesting to generate point mutants that specifically disrupt the interaction. AlphaFold3 allows identification of interaction interfaces between proteins facilitating the identification of target residues. Finally, as the availability of Cu and Fe greatly varied across geological times, it would also be interesting to consider the interactions between Cu and Fe homeostasis from an evolutionally point of view, comparing Arabidopsis with early plants and algae.
